# West Nile Virus Antibodies in Wild Birds, Morocco, 2008

**DOI:** 10.3201/eid1510.090340

**Published:** 2009-10

**Authors:** Jordi Figuerola, Riad E. Baouab, Ramon Soriguer, Ouafaa Fassi-Fihri, Francisco Llorente, Miguel Angel Jímenez-Clavero

**Affiliations:** Consejo Superior de Investigaciones Científicas, Seville, Spain (J. Figuerola, R. Soriguer); Université Mohammed V, Rabat, Morocco (R.E. Baouab); Institut Agronomique et Vétérinaire Hassan II, Rabat (O.Fassi-Fihri); Instituto Nacional de Investigación y Tecnología Agraria y Alimentaria, Valdeolmos, Spain (F. Llorente, M.A. Jímenez-Clavero)

**Keywords:** Flavivirus, West Nile virus, arboviruses, viruses, Morocco, birds, dispatch

## Abstract

To determine circulation of West Nile virus (WNV) during nonepidemic times, we serosurveyed wild birds of Morocco in 2008. We found antibodies against WNV in 12 (3.5%) birds, against Usutu virus in 1 (0.3%), and against both in 2 (0.6%). High WNV prevalence among juvenile birds suggests local virus circulation among resident birds.

In the Mediterranean basin, West Nile virus (WNV) causes sporadic disease outbreaks, which usually affect a low number of humans and animals, after which long periods without virus circulation occur. This pattern has occurred in France (outbreaks in 2000, 2003, 2004, and 2006), Italy (1998 and 2008), Algeria (1994), Tunisia (1997 and 2003), Morocco (1996 and 2003), Romania (1996–2000), and Israel (1998–2000) (*1*; www.oie.int/wahis/public.php?page=home). This finding has led to the hypothesis that the virus is absent from Europe and North Africa and periodically seeded into different places by infected migratory birds. An alternative hypothesis is that the virus can remain silent, circulating in a sylvatic enzootic bird–mosquito cycle and only under appropriate conditions causing new outbreaks in humans and horses (*2*). To test these 2 hypotheses, research under nonepidemic conditions is needed.

During the summer of 1996, WNV outbreaks caused the death of 42 horses and 1 human (*3*); during 2003, a total of 5 horses died from WNV infection (*4*). To determine circulation of the virus during a nonepidemic year, we conducted a serosurvey of wild birds in Morocco in 2008.

## The Study

From June through July 2008, we captured wild birds during 2 periods of 6 days each in Sidi Allal Tazi, Sidi Kacem Province (34°31′8′′N, 6°14′48′′W), ≈40 km northeast of Kenitra. The area is dominated by rice fields flooded from regulated channels from the Sebou River. Each captured bird was marked with a numbered metal ring; when possible, age was determined according to plumage characteristics. Blood was taken from the jugular vein and allowed to clot at ambient temperature. The blood was then centrifuged (10 min at 6,000 rpm), and the serum was stored in liquid nitrogen and transported to a deep freezer (–80°C) in the laboratory.

Neutralizing antibody titers against WNV (strain Spain/2007 GE-1B/b) and Usutu virus (USUV) (SAAR1776) were determined by using a micro virus-neutralization test as described previously (*5*). We used USUV as a control for WNV antibody specificity. Serum samples were inactivated at 56°C for 30 min before analysis. Dilutions of test serum (25 μL) were incubated with one hundred 50% tissue culture infective doses of the virus in the same volume (25 μL) for 1 h at 37°C in modified Eagle medium (*5*), after which 50 μL of a suspension (2 × 10^4^ cells/mL) of Vero cells plus fetal calf serum was added to the same medium to reach a final concentration of 5%. The mixture was further incubated for 6–7 days at 37°C until cytopathic effects were observed in control wells containing ten 50% tissue culture infective doses of virus. Samples were titrated by analyzing serial serum dilutions from 1:10 to 1:640. Only samples that showed neutralization (absence of cytopathic effect) at dilutions 1:>20 were considered positive. Samples that showed neutralization at the same dilutions were scored as positive for flavivirus but not conclusive for WNV and USUV. Controls for cytotoxicity in the absence of virus were included for every sample at a dilution of 1:10. Cytotoxicity prevented determination of neutralizing antibodies in 1 sample, which was therefore excluded from the analysis. A bird was considered to have seroconverted if it was seronegative at the time of first capture and seropositive (titer increase by at least 4-fold) at the time of recapture (*6*).

We analyzed 360 samples from 346 birds (Table). Neutralizing antibodies against WNV were found in 12 (3.5%) newly captured birds, against USUV in 1 (0.3%), and against both in 2 (0.6%). Positive results were obtained for 3 species. The highest prevalence was found among blackbirds (*Turdus merula*); neutralizing antibodies against WNV were found in 6 (19.3%) blackbirds, against flavivirus in 2 (6.5%), and against USUV in 1 (3.5%). Prevalence of WNV neutralizing antibodies among house sparrows (*Passer domesticus*) was much lower (2.2%). Additionally, 1 Cetti’s warbler (*Cettia cetti*) was negative for WNV neutralizing antibodies in June but had seroconverted by the time of recapture in July. Of the 13 additional birds sampled twice, 10 were negative for antibodies in both samples and 3 were positive in both samples. Prevalence of antibodies was significantly higher among adult than among juvenile (<1 year of age) blackbirds (χ^2^ = 8.22, 1 df, p = 0.004) but not among house sparrows (χ^2^ = 0.99, 1 df, p = 0.32).

**Table:** Number of wild birds with antibody titers against West Nile virus, by species, Morocco, 2008*

Species	Titer, no. birds (no. juveniles)						
	0	20	40	80	160	320	640
*Acrocephalus scirpaceus*	1 (1)	0	0	0	0	0	0
*Alcedo atthis*	4 (1)	0	0	0	0	0	0
*Asio otus*	1 (1)	0	0	0	0	0	0
*Carduelis carduelis*	9 (3)	0	0	0	0	0	0
*Carduelis chloris*	12 (2)	0	0	0	0	0	0
*Cettia cetti*	19 (4)	0	0	0	0	0	0
*Hippolais pallida*	49 (17)	0	0	0	0	0	0
*Hippolais polyglotta*	5 (2)	0	0	0	0	0	0
*Luscinia megarhynchos*	4 (2)	0	0	0	0	0	0
*Oriolus oriolus*	1	0	0	0	0	0	0
*Passer domesticus*	175 (109)	1	1	2 (1)	0	0	0
*Pycnonotus barbatus*	3	0	0	0	0	0	0
*Serinus serinus*	9 (3)	0	0	0	0	0	0
*Sturnus vulgaris*	1 (1)	0	0	0	0	0	0
*Sylvia melanocephala*	18 (7)	0	0	0	0	0	0
*Turdus merula*	23 (18)	4 (1)	1	1	0	0	2

*Captured in June (n = 197) and July (n = 149 + 14 recaptured birds first sampled in June); only data for first capture are shown.

## Conclusions

Antibodies in juvenile birds provide evidence of circulation of WNV and USUV in 2008 in the study area (maximum titers from neutralization tests were 80 for WNV and 160 for USUV). Because antibodies are maternally transmitted for only 2–4 weeks after birth (*7*,*8*), these high antibody titers suggest direct exposure to the virus during the summer of 2008. In addition, high titers (640) in 2 adult birds suggest recent circulation of WNV in the area. Blackbirds and house sparrows have short life spans; annual survival is estimated to be <50% (*9*). Consequently, antibodies in 58.3% of adult blackbirds cannot be explained by the exposure of these individual birds to WNV in 2003 (of 100 alive in 2003, <3 would be expected to be alive in 2008). All birds with neutralizing antibodies were members of nonmigratory (resident) species. These results suggest that WNV was present in Morocco in 2008 without resulting in disease outbreaks among humans or horses, as opposed to in 1996 and 2003, when cases did occur. The high prevalence of antibodies among adult birds also suggests that substantial circulation of the virus may have occurred during the previous season or seasons.

Although in recent years USUV circulation has been restricted to western Europe, especially in blackbirds (*10*,*11*), we did find 1 USUV-seropositive bird in Morocco. A strain of USUV was isolated from mosquitoes in Spain in 2006 (*12*), and serologic evidence of its circulation in Italy in 2007 has been found (*13*), indicating that the virus may have been circulating undetected in southern Europe. Our results suggest similar circulation in Morocco.

As evidence for silent circulation of WNV and USUV in the Mediterranean area (e.g., Spain, Czech Republic, or Italy) accumulates, the need to understand the ecologic factors related to virus circulation and the conditions leading to disease outbreaks increases (*5*,*11*,*14*). Factors may include strain characteristics, environmental conditions favoring virus amplification, or ecologic conditions favoring spillover to humans or horses, e.g., changes in mosquito feeding behavior increasing virus transmission from birds to humans (*15*). Long-term active monitoring programs that facilitate the understanding of virus circulation under nonepidemic conditions are needed.
